# Downregulation of STAT3, β-Catenin, and Notch-1 by Single and Combinations of siRNA Treatment Enhance Chemosensitivity of Wild Type and Doxorubicin Resistant MCF7 Breast Cancer Cells to Doxorubicin

**DOI:** 10.3390/ijms20153696

**Published:** 2019-07-28

**Authors:** Walhan Alshaer, Dana A. Alqudah, Suha Wehaibi, Duaa Abuarqoub, Malek Zihlif, Ma’mon M. Hatmal, Abdalla Awidi

**Affiliations:** 1 Cell Therapy Center, The University of Jordan, Amman 11942, Jordan; 2 Department of Pharmacology, Faculty of Medicine, The University of Jordan, Amman 11942, Jordan; 3 Department of Medical Laboratory Sciences, Faculty of Applied Health Sciences, Hashemite University, Zarqa 13133, Jordan; 4 Department of Hematology and Oncology, Jordan University Hospital, The University of Jordan, Amman 11942, Jordan

**Keywords:** breast cancer, siRNA, Notch-1, STAT3, β-catenin, doxorubicin, drug-resistance

## Abstract

Combinatorial therapeutic strategies using siRNA and small molecules to eradicate tumors are emerging. Targeting multiple signaling pathways decreases the chances of cancer cells switching and adapting new signaling processes that may occur when using a single therapeutic modality. Aberrant functioning of Notch-1, Wnt/β-catenin, and STAT3 proteins and their crosstalk signaling pathways have been found to be involved in tumor survival, drug resistance, and relapse. In the current study, we describe a therapeutic potential of single and combinations of siRNA designed for silencing Notch-1, Wnt/β-catenin, and STAT3 in MCF7_DoxS (wild type) and MCF7_DoxR (doxorubicin resistant) breast cancer cells. The MCF7_DoxR cells were developed through treatment with a gradual increase in doxorubicin concentration, the expression of targeted genes was investigated, and the expression profiling of CD44/CD24 of the MCF7_DoxS and MCF7_DoxR cells were detected by flow cytometry. Both MCF7_DoxS and MCF7_DoxR breast cancer cells were treated with single and combinations of siRNA to investigate synergism and were analyzed for their effect on cell proliferation with and without doxorubicin treatment. The finding of this study showed the overexpression of targeted genes and the enrichment of the CD44^−^/CD24^+^ phenotype in MCF7_DoxR cells when compared to MCF7_DoxS cells. In both cell lines, the gene silencing efficacy showed a synergistic effect when combining STAT3/Notch-1 and STAT3/Notch-1/β-catenin siRNA. Interestingly, the chemosensitivity of MCF7_DoxS and MCF7_DoxR cells to doxorubicin was increased when combined with siRNA treatment. Our study shows the possibility of using single and combinations of siRNA to enhance the chemosensitivity of cancer cells to conventional antitumor chemotherapy.

## 1. Introduction

Cancer remains one of the most frequent causes of morbidity and mortality worldwide [1]. In women, breast cancer is the most common and accounts for 25% of newly diagnosed malignancies [2]. The term cancer is described as a group of heterogeneous diseases caused by multiple genetic alterations that provoke complex network interactions among tumor cells and their surrounding niche, which is marked by the uncontrolled growth of cells [3]. During the last decades, the emerging molecular understanding of tumor biology and the mechanisms of drug resistance have afforded several successful therapeutic approaches [4]. For instance, the efficacy of antitumor chemotherapeutics has been improved using different strategies such as the development of targeted therapies and the engineering of drug delivery systems that can deliver payloads into tumor tissues [5,6]. However, these modalities are challenged by tumor heterogeneity and acquired drug resistance. As in many cancers, targeting individual signaling pathways may fail to eradicate tumors due to the capability of tumor cells to switch and adapt to new signaling pathways and mechanisms. Therefore, an important modality has been introduced by using combination therapy, which targets the multiple pathways involved in cancer progression [7]. Combinations of multiple chemotherapeutics, chemotherapeutics with radiation, and chemotherapeutics with therapeutic macromolecules have been successfully applied for the treatment of different types of cancers [8]. Recently, nucleic acid based therapeutics such as small interfering RNA (siRNA), micro RNA (miRNA), antisense oligonucleotides (AON), aptamers, small hairpin RNA (shRNA), and plasmid DNA (pDNA) have shown potent promises for cancer treatment [9].

siRNA has afforded a promising therapeutic approach by targeting specific genes involved in the pathogenesis of different diseases such as neurodegenerative diseases, pathogenic infections, and cancer [10,11]. Moreover, siRNA can be used to target single and multiple signaling pathways involved in disease progression. In breast cancer, abnormal functioning of different signaling pathways such as HER2, PI3K/AKT, TP53, MAPK, mTOR, Notch-1, Hedgehog, Wnt/β-catenin, and STAT3 have been found to be involved in tumorigenesis, tumor maintenance, drug resistance, and relapse [12,13]. Since these signaling pathways are unable to function separately, the crosstalk among them is very important for their functionality, which enhances the complexity of the disease. Therefore, developing combinations of drug regimens such as siRNA may provide more effective therapeutic outcomes. Notably, the crosstalk between the Notch-1, Wnt/β-catenin, and STAT3 signaling pathways has been reported in breast cancers and breast cancer stem cells [7]. Several reports have described the therapeutic potency of knocking down Notch-1, STAT3, and β-catenin proteins in breast cancer using siRNA [14,15,16]. In breast cancer, the expression of CD44 and CD24 has been linked to the identification of cancer stem cells, therapeutic responses, and the invasive behavior of tumor cells. For example, triple negative breast cancer cells that overexpress CD24 are more sensitive for doxorubicin and resistant to docetaxel [17]. Moreover, the downregulation of CD44 in breast cancer cells showed a higher sensitivity to doxorubicin [18]. Therefore, understanding the changes in the expression patterns of CD44 and CD24 in breast cancer after chemotherapy treatment is important for greater understanding of the clinicopathological properties of breast cancer.

Cancer cells may become unresponsive to several unrelated chemotherapeutic agents after long time exposure to a single drug, which is defined as multidrug resistance (MDR). For example, doxorubicin, an anthracycline antibiotic [19], is one of the most commonly used chemotherapeutic agents for the treatment of different cancers [20]. In breast cancer, the response rates are 43% in patients receiving doxorubicin for the first time, while the rates can decrease to 28% in patients previously treated with doxorubicin, indicating the development of resistance to doxorubicin among treated patients [21].

Although several studies have shown therapeutic efficacy by targeting Notch-1, β-catenin, and STAT3 using single siRNA modality, combinatorial siRNA therapy against these targets in wild type and drug resistant cancer cells have not been previously described. The aberrant functions of these targets are involved in tumorigenesis by deregulating signaling processes that control cell proliferation, epithelial-mesenchymal transmission (EMT), angiogenesis, apoptosis, metastasis, drug resistance, and stem cell activity. Therefore, in the current study, we developed a doxorubicin resistant MCF7 breast cancer cell line using a systematic increase in doxorubicin concentration. The MCF7 resistance to doxorubicin was confirmed by performing the viability assay and measuring the expression of multidrug-resistance related-genes. Next, we investigated the expression status of the Notch-1, β-catenin, STAT3, CD44, and CD24 markers in doxorubicin resistantMCF7 breast cancer cells (MCF7_DoxR) compared to MCF7 parental cells (MCF7_DoxS). Furthermore, both MCF7_DoxS and MCF7_DoxR cells were treated with single and combinations of siRNA to investigate the gene silencing efficacy and synergism of different siRNA treatments against Notch-1, β-catenin, and STAT3. Moreover, the chemosensitivity of MCF7_DoxS and MCF7_DoxR cells to doxorubicin was monitored in combination with siRNA treatment. The current work provides new insights toward developing more potent combinatorial therapeutic strategies using siRNA and small molecules to eradicate tumors.

## 2. Results and Discussion

### 2.1. Induction of Doxorubicin Resistant MCF7 Cells (MCF7_DoxR)

**Figure 1:**
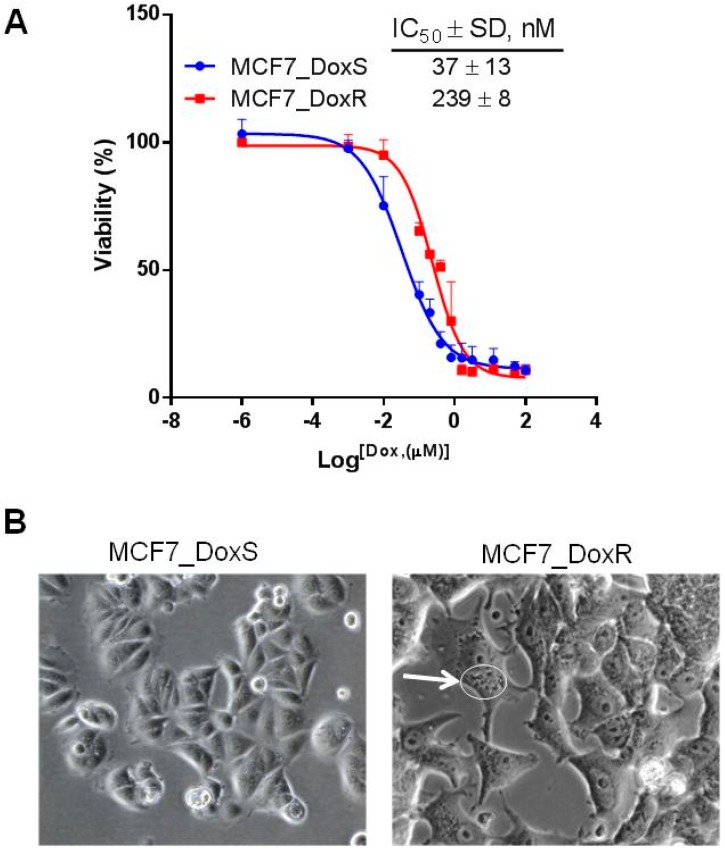
The development of doxorubicin resistance MCF7 cells (MCF7_DoxR). (**A**) Cell viability was measured using MTT assay to determine the IC_50_ (nM) of doxorubicin in MCF7_DoxR and MCF7_DoxScells after treatment with different concentrations of doxorubicin for 72 h. (**B**) The morphological appearance of MCF7 cells (20×) treated with doxorubicin (100 nM); the MCF7_DoxR contained multi-nucleated cytoplasm with large vesicles (white arrow/circle).

The capability of tumor cells to develop resistance to chemotherapies is a major challenge in tumor eradication. Therefore, understanding the molecular mechanisms behind the drug resistance phenomenon is crucial for the development of effective treatments for cancer. In the current work, the MCF7 breast cancer cell line was gradually treated with increased concentrations of doxorubicin starting at 10 nM and reaching a maximum of 100 nM. At each doxorubicin concentration, cells were maintained for six weeks in a given concentration to tolerate doxorubicin and gain resistance. The degree of resistance was confirmed by measuring the cell viability using the 3-[4,5-dimethylthiazol-2-yl]-2,5-diphenyltetrazolium bromide (MTT) assay. The IC_50_ of MCF7_DoxR was 239 ± 8 nM compared to 37 ± 13 nM in the MCF7_DoxS parental cells (Figure 1A). The degree of resistance is evaluated in terms of resistance index (R), which is calculated according to the relation: R = IC_50_ resistant cells/IC_50_ sensitive cells. Therefore, the relative resistance of MCF7_DoxR compared to the MCF7_DoxS control cells was significantly higher by ~6 folds (*p* < 0.0001). The morphological changes of the MCF7_DoxR cells after treatment with doxorubicin showed large multinucleated cells (MNCs) with large vesicles in the cytoplasm (Figure 1B). MNCs commonly appear in cancer cell lines and human cancer tissues and have been characterized as highly resistant to chemotherapy and have the capability of producing clonal, orthotopic, and metastatic tumors in vivo [22,23].

### 2.2. The Expression of Multidrug Resistant-Related Genes in MCF7_DoxR 

**Figure 2:**
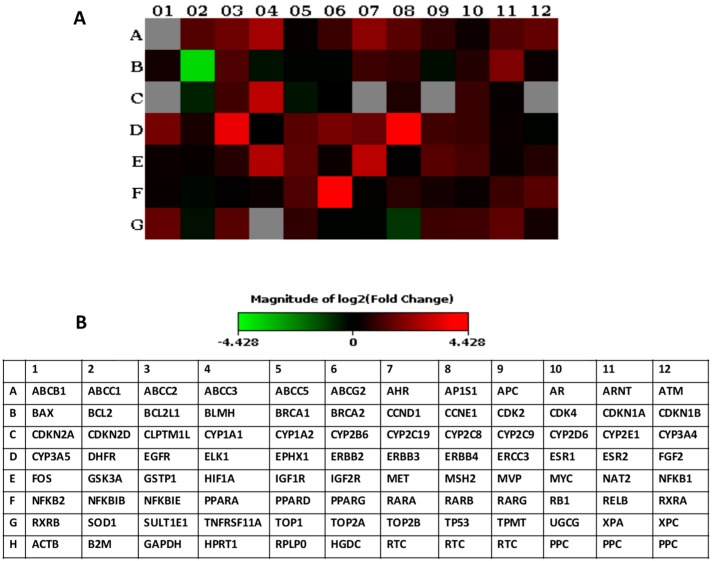
Multidrug resistant-related genes expression analyzed by RT2 profiler PCR array. (**A**) Heat map provides a visualization of the fold changes in the multidrug resistant-related genes expression in the MCF7_DoxR cells compared to the MCF7_DoxS cells. (**B**) Table showing the multidrug resistant-related genes used in the RT2 profiler PCR array experiments. HPRT1, B2M, and ACTB were used as housekeeping genes.

To confirm the employment of multidrug resistance mechanisms in MCF7_DoxR, the expression of multidrug resistant-related genes was explored using a Q-PCR array (Figure 2A,B) [22]. The maintenance of MCF7 cell cultures for a long time in vitro may induce different expression profiles for multidrug resistant-related genes, which is considered as an important issue when developing proper models for comparison. Therefore, both MCF7_DoxR and MCF7_DoxS cells were cultured under the same conditions including culturing medium, incubation times, and passage number. Interestingly, the upregulated genes in the MCF7_DoxR cells observed in our study lay within five important drug resistance-related mechanisms namely: drug efflux, drug inactivation, DNA damage repair, cell cycle and cell death inhibition, and growth factor receptors (Table 1).

### 2.3. Expression of Notch-1, STAT3, and β-Catenin in MCF7_DoxS and MCF7_DoxR Cells.

**Figure 3:**
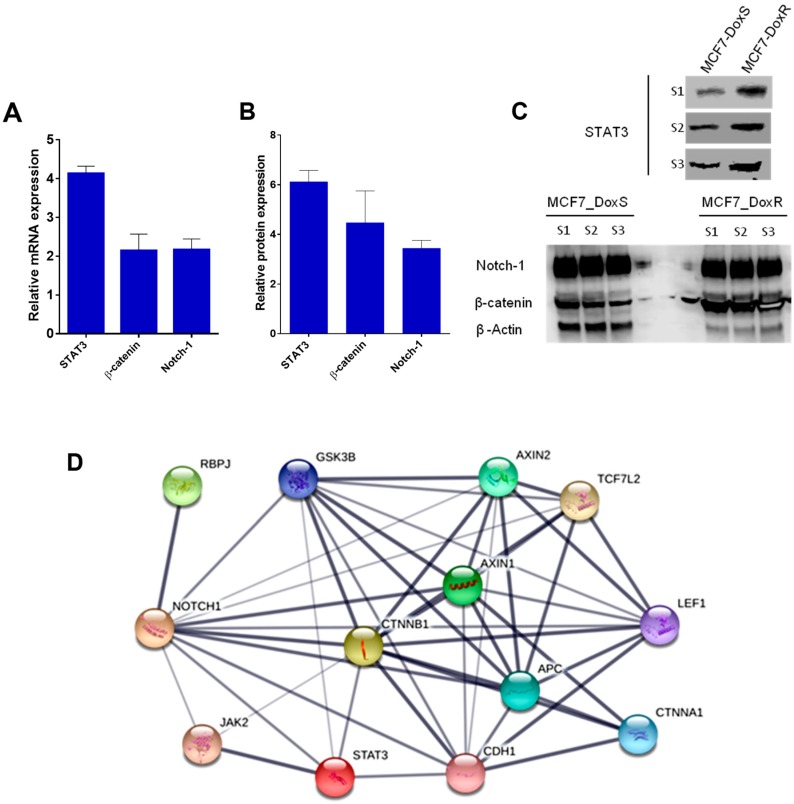
Expression of targeted genes. (**A**) The fold change in the mRNA expression of STAT3, β-catenin, and Notch-1 in MCF7_DoxR cells compared to the MCF7_DoxS cells (the 18srRNA gene was used as a housekeeping gene for Q-PCR). (**B**) The fold change in the protein expression of STAT3, β-catenin, and Notch-1 in MCF7_DoxR cells compared to the MCF7_DoxS cells. (**C**) The expression of STAT3, β-catenin, and Notch-1 proteins analyzed by WB (S1, S2, and S3 stands for three different samples). (**D**) STRING analysis of protein–protein interactions of the STAT3, β-catenin, and Notch-1 proteins, upregulated in MCF7_DoxR cells compared with MCF7_DoxS cells (thickness of edges indicates confidence) [39]. Data represent the average of three independent experiments ± SD.

To investigate changes in the expression of STAT3, β-catenin, and Notch-1 targets at both the mRNA and protein levels as a result of doxorubicin exposure, Q-PCR and Western blot were performed. Interestingly, the analysis of the Q-PCR results showed a significant upregulation in the mRNA expression of STAT3 (4.1 ± 0.1 folds), β-catenin (2.1 ± 0.3 folds), and Notch-1 (2.2 ± 0.2 folds) in MCF7_DoxR compared to the MCF7_DoxS cells (Figure 3A). The Western blot analysis showed upregulation in the protein expression of STAT3 (6.1 ± 0.8 folds), β-catenin (4.5 ± 2.2 folds), and Notch-1 (3.4 ± 0.6 folds) in MCF7_DoxR compared to the MCF7_DoxS cells (Figure 3B,C). Thus, the overexpression of the targeted genes in MCF7_DoxR compared to MCF7_DoxS cells highlights the importance of the crosstalk that can occur among these genes, which might have an important role in the development of doxorubicin resistance in MCF7_DoxR cells. A simple assessment of STAT3, β-catenin, and Notch-1 network interactions using the STRING database predicted the possible direct and indirect interactions among these genes (Figure 3D).

Our results are in agreement with previous reports that have described the roles of STAT3, β-catenin, and Notch-1 in tumorigenesis and drug resistance. STAT3 is located at many connecting points of different oncogenic signaling pathways. Although normal cells can tolerate the disruption of STAT3 with little toxicity due to the redundancies in the normal signaling pathways, cancer cells employ alterations in the activity and functionality of STAT3 that enhance tumor progression. Thus, STAT3 is considered to be an important therapeutic target to prevent tumor progression [24]. Several studies have claimed the roles of the targets in inducing drug-resistance and enhancing tumorigenesis and relapse. For example, the loss of adenomatous polyposis coli (APC) functionality has been reported to be associated with cisplatin and doxorubicin resistance in breast cancer [25]. This resistance mechanism has been explained by the overexpression of EGFR, which activates the STAT3 protein that upregulates the expression of multidrug resistance protein 1 (MDR1) [26]. Furthermore, the irregular activation of the Wnt/β-catenin signaling pathway can upregulate the formation of abnormal proteins and enhance tumor proliferation [27,28]. The inactivation of the Wnt/β-catenin pathway through the silencing of β-catenin activity using siRNA was able to reverse the drug resistance of cancer cells and inhibit their proliferation rate [29,30]. Moreover, the activation of β-catenin can enhance the expression of the MDR1 (P-glycoprotein/P-gp) gene and the anti-apoptotic gene Bcl-xL, which can cause chemoresistance to doxorubicin and etoposide in MCF7 breast cancer cells [31]. Aberrant Notch-1 signaling has been linked to tumor progression and the reduction of overall survival rates. Additionally, the crosstalk between Notch-1 signaling and other oncogenic pathways such as Wnt/β-catenin, NF-kB, Ras, and Akt have shown to have an important impact on the formation and aggressiveness of tumors [32,33,34]. Notch-1, in particular, has been proven to be involved in the carcinogenesis of breast cancer [35], and the maintenance of the malignant phenotype of transformed cells [36]. Moreover, the overexpression of Notch-1 induces mammary tumors in mice [37]. In addition, Notch-1 can induce the expression of multidrug resistance-associated protein 1 (ABCC1/MRP1) through the regulation of transcription factor centromere-binding protein 1 (CBF1) in MCF7 breast cancer cells [38].

### 2.4. Expression of CD44/CD24 Surface Markers 

**Figure 4:**
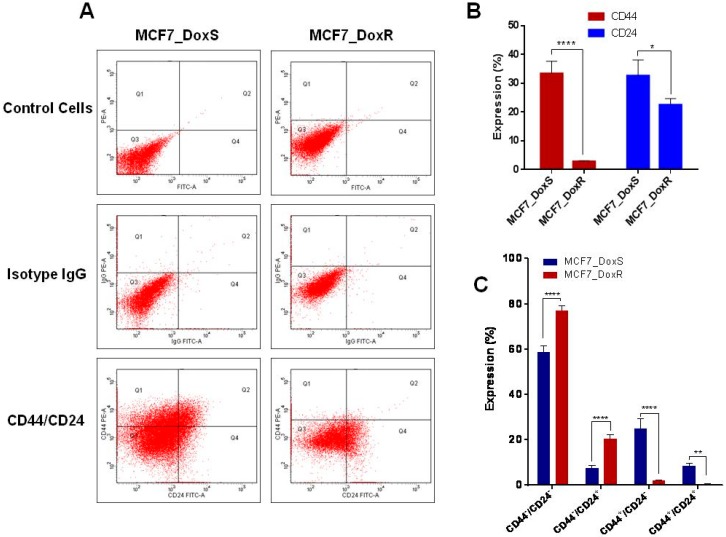
Flow cytometric analysis for CD44 and CD24 expression by MCF7_DoxS control cells and the MCF7_DoxR cells. (**A**) Dot plot analysis for MCF7_DoxS and MCF7_DoxR cells labeled with anti-CD24-FITC and anti-CD44-PE. (**B**) The expression of individual CD44 and CD24. (**C**) The expression phenotype. The expression of markers was compared to their isotype controls. The data represent the average of three independent experiments ± SD. Statistical significance: Student’s t test, * indicates *p* < 0.05, ** indicates *p* < 0.01, **** indicates *p* < 0.0001.

Breast cancer displays high tumor heterogeneity and different phenotypes that drive tumor progression, metastasis, recurrence, and drug resistance. For example, breast cancer cells with the phenotype CD44^+^/CD24^−/low^ have been characterized as having stem cell-like properties that can initiate tumors in xenograft models associated with chemo-resistance [40]. On the other hand, several studies have revealed that the CD44^−^/CD24^+^ phenotype is associated with a worse prognosis. The expression of CD44 and CD24 is associated with prognostic data and therapeutic outcomes [41,42]. The upregulation of STAT3, β-catenin, and Notch-1in MCF7_DoxR cells has raised the question about the status of CD44/CD24 breast cancer stem cell markers and if the MCF7_DoxR cells show a higher enrichment of breast cancer stem cell populations. Therefore, the expression of CD44 and CD24 was evaluated in this study (Figure 4A,B). Interestingly, our data showed a significant decrease in the expression of CD44 (*p* < 0.0001) and CD24 (*p* < 0.05) in MCF7_DoxR compared to the MCF7_DoxS parental cells. However, when both markers are taken together, a significant increase in the CD44^−^/CD24^+^ population was observed in the MCF7_DoxR (20.3 ± 1.9) compared to the MCF7_DoxS (7.6 ± 1.4) parental cells (*p* < 0.0001) (Figure 4C). Al-Hajj et al. and other reports have described the association of CD44^+^/CD24^−^/low population in breast tumors with cancer stem cell properties, as this is responsible for drug resistance and tumor relapse [40,41,42]. However, several studies have been performed to investigate the clinical and prognostic value of CD44 and CD24 expression in clinical samples, which have shown that the CD44^−^/CD24^+^phenotype is associated with poor prognosis compared to the CD44^+^/CD24^−/low^ phenotype, which showed better prognosis [43,44]. Moreover, the expression of CD24 has been associated with a higher tumor grade and more aggressive behavior. In contrast, CD44 positivity has been associated with a better prognosis [45]. Such results are consistent with our findings and provide new insights into the development of doxorubicin resistant cancer cell lines in vitro, mimicking the clinical situation for the use of anticancer therapeutics. Moreover, doxorubicin resistant cells may show different gene expression profiles in relation to the doxorubicin dose and time of treatment and maintenance of cells [46].

### 2.5. Silencing of Notch-1, STAT3, and β-Catenin in MCF7_DoxS and MCF7_DoxR Cells Affects Cell Proliferation

**Figure 5:**
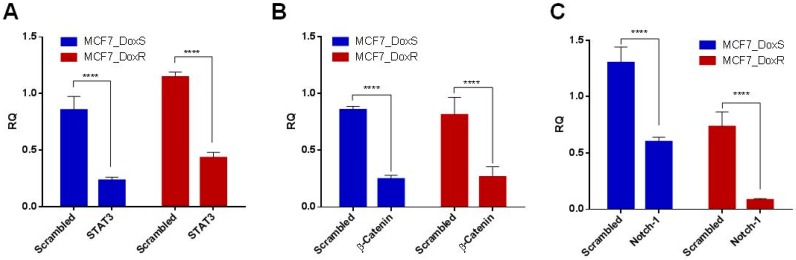
Gene-silencing effect of each siRNA. The mRNA expression of the targeted genes in MCF7_DoxR and MCF7_DoxS cells after transfection with 50 nM of (**A**) STAT3; (**B**) β-catenin; and (**C**) Notch-1 siRNA for 72 h. Scrambled siRNA was used as a negative control for comparison. The 18srRNA gene was used as a housekeeping gene for Q-PCR. The data represent the average of three independent experiments ± SD. Statistical significance: Student’s *t*-test, **** indicates *p* < 0.0001.

To evaluate the silencing efficacy of siRNA after confirming the overexpression of targeted genes, Q-PCR was performed after the treatment of 50 nM for each siRNA. In both cell lines, the results showed a significant reduction in the mRNA expression of each targeted gene compared to the scrambled siRNA (*p* < 0.0001) (Figure 5A–C). 

**Figure 6:**
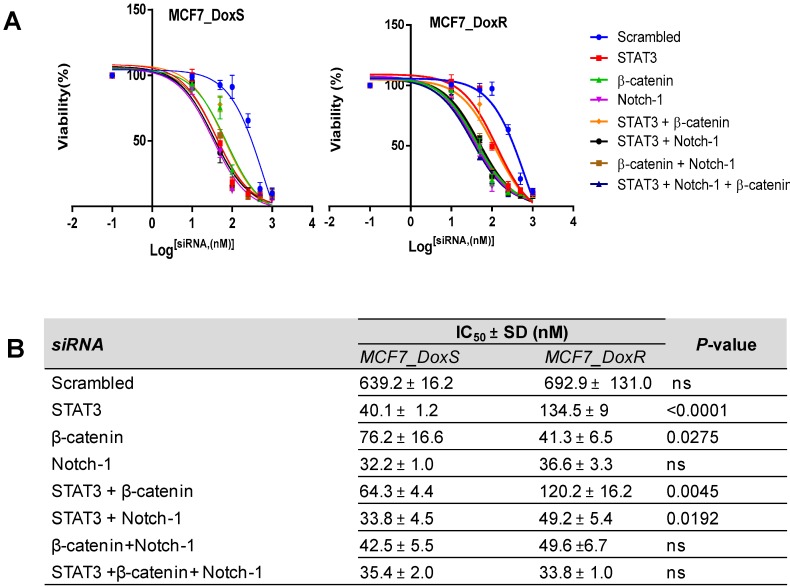
Dose–response curves and the IC_50_ values after siRNA treatment. The cell lines were treated with different concentrations of single and combinations of siRNAfor 72 h to assess the anti-proliferative effect on MCF7_DoxS and MCF7_DoxR cells. (**A**) The dose–response curves and (**B**) the IC_50_ values of single and combinations of siRNA in MCF7_DoxSand MCF7_DoxR cells. Scrambled siRNA was used as a negative control for comparison. All IC_50_ values represent the average of three independent experiments ± SD with four replicates per siRNA concentration for each experiment. Statistical significance: Student’s *t*-test, *p* < 0.05 indicates a significant difference, ns: not significant.

Further confirming the specific-gene silencing of siRNA molecules, the effect of siRNA on the viability of both MC7-DoxR and MCF7-DoxS cells was evaluated using the viability assay. Cells were treated with different concentrations of each single and combination of siRNA for 72 h and a scrambled siRNA was used as a negative control to compare nonspecific toxicity. Remarkably, all single and combinations of siRNA showed a decrease in the cell viability in a dose-dependent manner (Figure 6A). The IC_50_ values of single and combinations of siRNA were determined (Figure 6B). Our findings showed a significant decrease in the IC_50_ values in MCF7_DoxR and their control cells MCF7_DoxS compared to the scrambled siRNA (*p* < 0.0001). Single siRNA treatments showed a different impact on the IC_50_ values for each cell line. Notch-1 siRNA showed no significant difference in the IC_50_ values for both MCF7-DoxR (36.6 ± 3.3) and their control cells MCF7-DoxS (32.2 ± 1.0). Interestingly, the IC_50_ of STAT3 siRNA was significantly lower in the MCF7-DoxS cells (40.1 ± 1.2) compared to the MCF7-DoxR cells (134.7 ± 9.0) (*p* < 0.0001). Meanwhile, β-catenin siRNA was able to induce a significant decrease in the IC_50_ value in MCF7-DoxR cells (41.3 ± 6.5) compared to MCF7-DoxS (76.2 ± 16.6) (*p* < 0.05). In the case of the siRNA combinations, all siRNA combinations showed close IC_50_ values in both cell lines except for the STAT3 and β-catenin combination, which was higher for both cell lines with a significant effect on the MCF7_DoxS cells (64.3 ± 4.4) compared to the MCF7_DoxR cells (120.2 ± 16.2) (*p* < 0.01). The STAT3 and Notch-1 combination was significantly lower in the MCF7_DoxS cells (33.8 ± 4.5) compared to the MCF7_DoxR cells (49.2 ± 5.4) (*p* < 0.05). There was no significant difference noticed for the IC_50_ STAT3 and β-catenin combination and STAT3 Notch-1, and β-catenin combination in both the MCF7_DoxS and MCF7_DoxR cells. 

Our results are in agreement with several reports that have described the antitumor effect of Notch-1, STAT3, and β-catenin downregulation. For example, the downregulation of the Notch-1 gene in MCF7 breast cancer cells showed a reduction in tumor cell proliferation through the induction of apoptosis, which may result from the inactivation of NF-κB signaling [14]. Moreover, the downregulation of Notch-1 using siRNA was found to enhance the chemosensitivity of cancer cells to docetaxel and doxorubicin [14]. STAT3 has been found to be constitutively active in different cancers including 40% of breast cancers. STAT3 regulates several genes that are involved in tumorigenesis through different signaling pathways such as the upregulation of cell proliferation via cyclin D, c-Myc, and the increased expression of anti-apoptotic proteins including Bcl-XL, Mcl-2, and surviving [47]. Knock down of STAT3 by siRNA was able to suppress tumor growth in vitro and in a xenograft model of human breast cancer through the induction of apoptosis [15]. More than 50% of breast cancers have activated Wnt/β-catenin signaling, which is associated with lower overall survival rates. In fact, atypical activation of β-catenin leads to the constitutive formation of the β-catenin/TCF complex, which activates the expression of target genes that are involved in tumor progression including c-Myc, cyclin-D1, and MMP-7. Moreover, β-catenin forms a complex with NF-κB and inhibits its function, leading to the inhibition of Fas expression, which is needed for proapoptotic events [48].

### 2.6. Combination Index

To investigate the possible synergistic effect following the treatment of both cell lines with combinations of siRNA, the combination index (CI) was calculated using Compusyn software. The calculation of CI showed a synergistic effect on MCF7_DoxR and their related control cells of MCF7_DoxSwhen treated with either a combination of STAT3, β-catenin, and Notch-1 siRNA or a combination of STAT3 and Notch-1, as the value of CI < 1 (Table 2).On the other hand, two different combinations, a combination of STAT3 and β-catenin, and β-catenin and Notch-1 showed an additive effect as CI~1. These results indicate the presence of crosstalk between the selected targets, and this crosstalk can regulate the expression and activity of the other genes at both the transcriptional and translational levels [49,50,51,52,53]. Thus, our data are in agreement with previous studies that have reported the presence of crosstalk between STAT3 and Notch-1 [37]. Notch-1 can upregulate IL6 via the JAK/STAT3 signaling pathway, which is involved in breast cancer survival and proliferation [52]. Moreover, the crosstalk between Notch-1 and β-catenin has been reported via activation of the Notch-1 by Wnt1/β-catenin pathway in human breast cancer [54]. Furthermore, STAT3 expression can upregulate the expression of β-catenin in breast cancers [49]. However, our findings indicated thatno synergistic effect was found when cells treated with either of the STAT3 and β-catenin, or β-catenin and Notch-1 siRNA combinations, and this is likely explained by cells relying on alternative signaling pathways for survival.

### 2.7. The IC_50_ of Doxorubicin Decreased after siRNA Treatment

**Figure 7:**
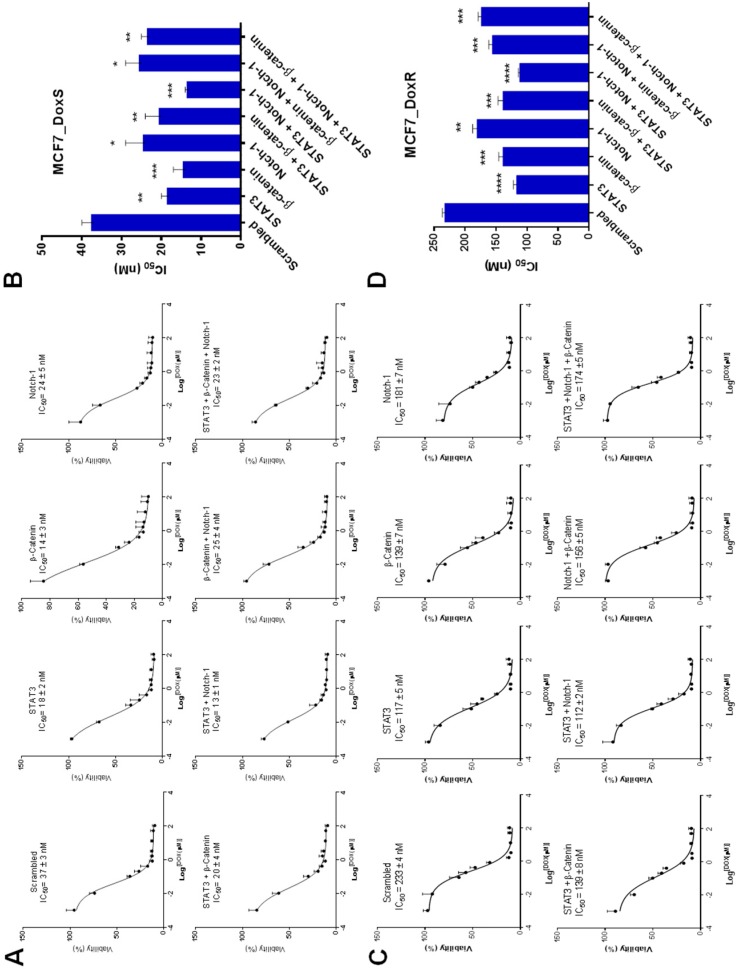
IC_50_ values after siRNA and doxorubicin treatment. Both MCF7_DoxS and MCF7_DoxR cells were treated with a fixed concentration of siRNA (50 nM) and different concentrations of doxorubicin for 72 h to assess the effect of siRNA and doxorubicin combinations on cell viability using the MTT assay. (**A**) The dose–response curves and (**B**) the IC_50_ values of doxorubicin combined with single and combinations of siRNA in MCF7_DoxS. (**C**) The dose–response curves and (**D**) the IC_50_ values of doxorubicin combined with single and combinations of siRNA in MCF7_DoxR. Scrambled siRNA was used as the negative control for comparison. All IC_50_ values represent the average of three independent experiments with four replicates per siRNA concentration for each experiment. Statistical significance: Student’s t test, * indicates *p* < 0.05, ** indicates *p* < 0.01, *** indicates *p* < 0.001, **** indicates *p* < 0.0001.

To investigate the effect of single and combinations of siRNA treatment on the chemosensitivity of MCF7_DoxR and their control cells MCF7_DoxS to doxorubicin treatment, both MCF7_DoxS and MCF7_DoxR cells were first treated with 50 nM of single and combinations of siRNA for 6 h followed by treatment with different concentrations of doxorubicin. The IC_50_ values of doxorubicin on MCF7_DoxR and their control cells MCF7_DoxS were calculated (Figure 7A,C). The results of the IC_50_ values indicated a significant increase in the chemosensitivity of both MCF7_DoxS and MCF7_DoxR cells to doxorubicin when treated with all single and combinations of siRNA compared to cells treated with scrambled siRNA (Figure 7B,D). This could be explained by the role of the selected targets in tumor proliferation and inducing drug resistance. Our findings showed that STAT3 expression was~2 folds higher in MCF7_DoxR cells when compared to β-catenin and Notch-1. This may explain why STAT3 siRNA alone or the STAT3 and Notch-1 siRNA combination showed higher sensitivity for Dox in MCF7_DoxR and MCF7_DoxS cells. Such findings can be explained by the dominating roles STAT3 and Notch-1 have in inducing Dox resistance in MCF7_DoxR cells. Generally, our results are consistent with several reports that have previously described the enhanced sensitivity of cancer cells to chemotherapy when combined with STAT3, β-catenin, and Notch-1 inhibitor treatment. For example, Zang et al. [14], demonstrated that the combination of Notch-1 downregulation with doxorubicin and docetaxel increased the cell growth inhibition in MDA-MB-231 and MCF7 cells by 50 or 70% by increasing the apoptotic cells when compared to chemotherapy alone. Moreover, Gariboldiet al. [55] reported the enhanced sensitivity of metastatic MDA-MB-213 breast cancer cells to doxorubicin when combined with STAT3 inhibition. In addition, Xu et al. [56] showed that triple negative breast cancer cells treated with β-catenin shRNA were more sensitive for doxorubicin and cisplatin chemotherapy. 

## 3. Materials and Methods 

### 3.1. Oligonucleotides

The Q-PCR primers were obtained from Integrated DNA Technologies (IDT, San Diego, CA, USA) and designed as follows: 18s rRNA reference gene forward: 5′-AGGAATTCCCAGTAAGTGCG-3′ and reverse: 5′-GCCTC ACTAAACCATCCAA -3′; STAT3 forward: 5′-ATCATAGGGACCTAGGGCGAG-3′, and reverse: 5′-TTTAATGGGCCACAACAGGG-3′; Notch-1 forward: 5′-CTGAATTTCACTG TGGGCGG -3′, and reverse: 5′-CCCCGCAGAGGGTTGTATTG-3′; β-catenin forward: 5′-CCATTCTGGTGCCACTACCA-3′, and reverse: 5′-CAGGGAACATAGCAGCTCGT-3′. The siRNA sequences were obtained from Integrated DNA Technologies (IDT, San Diego, CA, USA) and designed as follows: Scrambled sense: 5′-UUCUCCGAACGUGUCACGU-3′ and antisense: 5′-ACGUGACACGUUCGGAGAA-3′; STAT3 sense: 5′-CCAAGUUCAUGGCCUUAGGUAG-3′, and antisense 5′-CU ACCUAAGGCCAUGAACUUGG-3′ [57];Notch-1 sense: 5′-UCGCAUUGACCA UUCAAACUGGUGG-3′ and antisense: 5′-CCACCAGUUUGAAUGGUGAAUGCGA-3′ [58]; β-catenin sense: 5′-CUCAGUCCUUCACUCAAGA-3′ and antisense: 5′-UCUUGAGUGAAG GACUGAG-3′ [59]. 

### 3.2. Cell Culture

The parental MCF7 breast cancer cell line was obtained from the American Type Culture Collection (ATCC, Manassas, VA, USA). MCF7 cells were cultured as an attached monolayer and maintained in RPMI 1640 medium (EuroClone, Milan, Italy) supplemented with10% (v/v) heat-inactivated fetal bovine serum (FBS) (EuroClone, Milan, Italy), 0.5% penicillin-streptomycin (EuroClone, Milan, Italy), and 2 mM L-glutamine. Cells were incubated at 37 °C in a 5% CO_2_ tissue culture incubator (Memmert, Schwabach, Germany).

### 3.3. Development of MCF7 Doxorubicin Resistant Cells (MCF7_DoxR)

Doxorubicin resistant cells were established by the gradual treatment of MCF7 cells with increased concentrations of doxorubicin (Ebewe, Mondsee, Austria) over a period of 14 months. As a starting point, 10 nM of doxorubicin was directly applied to MCF7 cells and allowed to grow to reach 80% confluence. Then, cells were sub-cultured regularly and the concentration of doxorubicin was increased by 10 nM each time until reaching a maximum concentration of 100 nM. Control MCF7 cells (MCF7_DoxS) were cultured in parallel with the MCF7_DoxR cells for comparison.

### 3.4. Array Q-PCR for Quantification of Multidrug Resistance Related-Genes Expression

RNA was extracted using the Trizol-hybrid method (Qiagen, Hilden, Germany). Then, cDNA was synthesized by converting 0.5 μg total RNA using the RT2 First Strand Kit^®^ (Qiagen, Hilden, Germany). A diluted cDNA aliquot was mixed with the RT2 SYBR^®^ green master mix of RT2 Profiler™ PCR Array of Human Cancer Drug Resistance kit^®^ (Qiagen, Hilden, Germany), according to the manufacturer’s instructions, and loaded into the 96-well array plate. Q-PCR reactions were performed using the CFX96 C1000 Touch thermal cycler (Bio-Rad, Hercules, CA, USA) with the following temperature setting: (i) 95 °C for 10 min, (ii) 40 cycles of 95 °C for 15 s, and (iii) 60 °C for 1 min. The data analysis was performed using the 2^−ΔΔCt^ method available from the SABiosciences company (Qiagen, Hilden, Germany) [60]. The data were normalized across all plates to the following housekeeping genes: Hypoxanthine phosphoribosyltransferase 1 (HPRT1), Beta-2-microglobulin (B2M), and actin beta (ACTB). The threshold cycle values of the control wells were all within the ranges recommended by the PCR array user manual.

### 3.5. Q-PCR for Quantification of STAT3, β-Catenin, and Notch-1 Expression

In order to determine the expression of the target genes STAT3, Notch-1, and β-catenin at the mRNA level, qPCR was performed. Both the MCF7_DoxS and MCF7_DoxR cells were lysed by the Trizol-hybrid method for RNA extraction (Qiagen, Hilden, Germany). The extracted RNA was quantified by a Nanodrop (Thermofisher, Waltham, MA, USA). To synthesize cDNA, 0.5 μg total RNA was reverse transcribed using the PrimeScript™ RT Master Mix (Takara, Dalina, China) using a T100™ Thermal cycler PCR instrument (Bio-Rad, Hercules, CA, USA). The Q-PCR reaction mix was prepared by mixing 2 μL of cDNA with 0.4μLof forward primer, 0.4μLof reverse primer, 7.2 μL of free nuclease water, and 10 μL of KAPA SYBR FAST qPCR Master Mix (Kapa, Wilmington, MA, USA). Q-PCR was performed by using a CFX96 C1000 Touch thermal cycler (Bio-Rad, Hercules, CA, USA) with the following temperature setting: (i) 95 °C for 2 min, (ii) 40 cycles 95 °C for 15 s and 64 °C for 30 s. 18S rRNA was used as a reference gene. Data were analyzed using the 2^−∆∆CT^ method [61]. A similar Q-PCR protocol was used to analyze the specific-gene silencing efficacy of each siRNA. Briefly, the MCF7_DoxS and MCF7_DoxR cells (5 × 10^5^ cells/well) were plated into 6-well plates and treated with 50 nM of different siRNA preparations. After 48 h of treatment, the mRNA of each gene was quantified and compared to the untreated cells and a scrambled siRNA control was used for the off-target effect. 

### 3.6. Western Blot

Cell pellets from both MCF7_DoxS and MCF7_DoxR cells were lysed in RIPA buffer (Thermofisher, Waltham, MA, USA) containing phosphatase and protease inhibitors for 30 min on ice. Protein concentrations were assayed using the BCA Protein Assay Kit (Abcam, Cambridge, UK). A total of 20 µg of proteins were boiled with laemmli sample buffer for 5 min at 95 °C. SDS-PAGE was performed with the Mini-PROTEAN TGX Precast gel (Bio-Rad, Hercules, CA, USA). Proteins were transferred to a PVDF membrane (Bio-Rad, Hercules, CA, USA). For immunodetection, membranes were blocked in TBST buffer (Tris-buffered saline, 0.1% Tween 20) containing 5% skimmed milk for 1 h at room temperature followed by incubation with primaryantibody dilutions overnight (4 °C) (anti-β-actin 1:2000, anti-β-catenin 1:5000, anti-Notch-1 1:1000, and anti-STAT-3 1:1000) (Abcam, Cambridge, UK). After washing, horse-radish peroxidase-coupled secondary antibody (anti-rabbit 1:5000) (Abcam, Cambridge, UK) was added for 1 h and the detection was carried out using a SuperSignal™ West Pico PLUS Chemiluminescent substrate (Thermofisher, Waltham, MA, USA)anda C-Digit Blot Scanner (LI-COR Biosciences, Lincoln, NE, USA) was used for imaging. Densitometric analysis was performed on the blots using ImageJ (Version 1.51w, National Institute of Health, MD, USA) [62].

### 3.7. Detection of CD44 and CD24 Expression

To identify the expression of CD44/CD24 markers from the MCF7_DoxS and MCF7_DoxR cells, a flow cytometry assay was performed. Both MCF7_DoxS and MCF7_DoxR cells were detached using StemProAccutase^®^ Cell Dissociation Reagent (Gibco, Waltham, MA, USA). MCF7_DoxS and MCF7_DoxR cells were washed twice with PBS (Gibco, Waltham, MA, USA) and 1 × 10^6^ cells/mL from each cell type were incubated with anti-CD44-PE (Biolegend, San Diego, CA, USA) and anti-CD24-FITC (Biolegend, San Diego, CA, USA) for 20 min in the dark at room temperature. Following that, cells were centrifuged at 300× *g* for 5 min and re-suspended in 300 µL PBS. PE- and FITC-IgG1 isotype controls (Biolegend, San Diego, CA, USA) were used for the nonspecific binding. The expression profiles were analyzed by a Fluorescein activated sorter FACS Canto II using FACS Diva 7 software (BD San Diego, CA, USA). 

### 3.8. Detection of Cell Viability by MTT Assay

#### 3.8.1. IC_50_ of Doxorubicin before siRNA Treatment

To determine the IC_50_ of doxorubicin for both the MCF7_DoxS and MCF_DoxR cells, a MTT assay was performed. Both MCF7_DoxS and MCF7_DoxR cells were seeded into 96-well plates (8 × 10^3^ cells/well) and allowed to attach for 24 h, then treated with different concentrations of doxorubicin and incubated for 72 h at 37 °C in a 5% CO_2_ incubator. After incubation, the old medium was replaced with 100 µL fresh medium and 15 µL of MTT [3-[4,5-dimethyl-2thiazolyl]-2,5-diphenyl-2H-tetrazolium bromide] (5 mg/mL) (Promega, Madison, WI, USA) was added to each well and the plates were incubated at 37 °C for three hours. Then, the reaction was stopped by the addition of 50 µL/well DMSO for 10 min. Optical density (O.D.) was measured at 570 nm using a Glomax plate reader (Promega, Madison, WI, USA).

#### 3.8.2. IC_50_ of siRNA Treatment

To determine the anti-proliferative effect of siRNA in both the MCF7_DoxS and MCF_DoxR, cells from each cell line were seeded into 96-well plates (8 × 10^3^ cells/well) and incubated at 37 °C in a 5% CO_2_ incubator for 24 h for attachment. Then, single and different combinations of siRNA were prepared as follows: Notch-1, STAT3, β-catenin, Notch-1+STAT3 (1:1 molar ratio), Notch-1 + β-catenin (1:1 molar ratio), STAT3 + β-catenin (1:1 molar ratio), and STAT3 + β-catenin + Notch-1 (1:1:1 molar ratio), and were complexed with Lipofectamine 3000 (Invitrogen, Carlsbad, CA, USA) for cell transfection. Both MCF7_DoxS and MCF7_DoxR cells were treated with different concentrations of siRNA formulations (10, 50, 100, 250, 500, and 1000 nM) in a serum-free medium for 6 h at 37 °C in a 5% CO_2_ incubator, followed by the addition of 10% FBS. After 72 h of incubation, the cell viability was detected using the same MTT assay as described above. Scrambled siRNA was used as a negative control for all siRNA formulations. 

#### 3.8.3. IC_50_ of Doxorubicin after siRNA Treatment (Chemosensitivity)

To investigate the effect of single and combinations of siRNA on doxorubicinIC_50_ values, both MCF7_DoxR and MCF7_DoxS cells were seeded into 96-well plates (8 × 10^3^ cells/well) and incubated at 37 °C in a 5% CO_2_ incubator for 24 h for attachment. Then, cells were treated with 50 nM of different siRNA formulations in a serum-free medium for 6 h at 37 °C in a 5% CO_2_ incubator. After incubation, the medium was replaced with cell culture medium containing serum and different concentrations of doxorubicinfor a total time of 72 h. After incubation, the cell viability was detected using the same MTT assay described above.

### 3.9. Combination Index

To determine the effect of using different combinations of siRNA complexes on MCF7_DoxR and their control cells MCF_DoxS, the combination index was calculated using Compusyn software (Version 1.0, Compusyn, Inc., Paramus, NJ, USA). Briefly, an isobologram analysis was performed using the Compusyn software program. Cells were treated with different combinations of siRNA either individually or in combinations of a1:1 molar ratio for 72 h followed by MTT assay analysis to determine the cell viability and combination index (CI). A CI of <1.0 indicates synergism, a CI of 1 indicates additive activity, and a CI > 1.0 indicates antagonism. For a single siRNA treatment, the potential synergy between combinations of siRNA was evaluated as described previously [63]. 

### 3.10. Statistical Analysis 

The statistical analyses were performed using the Student’s *t*-test. All values were expressed as mean ± SD and the significant difference as considered when the *p*-value was less than 0.05. 

## 4. Conclusions

In this study, we developed a doxorubicin resistant MCF7 breast cancer cell line, followed by study of the expression status of important oncogenes including STAT3, β-catenin, and Notch-1. All three oncogenes were found to be overexpressed in the doxorubicin resistant MCF7 cells (MCF7_DoxR) compared to the wild-type MCF7 cells (MCF7_DoxS), indicating the important role of these proteins in drug resistance and tumor maintenance. Moreover, the expression of CD44/CD24 stemness surface markers was significantly reduced in the MCF7_DoxR cells compared to the MCF7_DoxS parental cells. Our data suggest that combinations of siRNA such as STAT3 and Notch-1 or STAT3, β-catenin, and Notch-1 can produce synergistic effects against MCF7_DoxS cells and MCF7_DoxR cells. Silencing these targets by using STAT3 siRNA or STAT3 and Notch-1 siRNA can enhance thechemosensitivityofMCF7_DoxS cells and MCF7_DoxR cells to doxorubicin. Altogether, our findings describe for the first time the therapeutic potential of the use of siRNA combinations to enhance chemosensitivity to doxorubicin in breast cancer.

## Figures and Tables

**Table 1:** The genes expression profiling of multidrug resistant-related genes in MCF7_DoxR compared to the MCF7_DoxS parental cells analyzed by RT2 profiler PCR array. A standard 2-fold change was used as arbitrary cut-off.

Mechanism		Fold Change
**Upregulated (≥2-folds)**		
Drug efflux	ABCC1	2.4
	ABCC2	3.0
	ABCC3	5.4
	MVP	2.5
Drug inactivation	CYP1A1	7.1
	CYP3A5	3.4
	AhR	4.1
	ARNT1	2.3
	SULT1E1	2.5
	EPHX1	2.4
Cell cycle and cell death inhibition	PPARG	14.5
	MET	6.8
	HIF1A	6.2
	CDKN1A	3.6
	RXRB	2.8
	IGF1R	2.6
	PPARD	2.2
	IGF1R	2.6
	XPA	4.6
Growth factors receptors axes	ERBB4	21.5
	EGFR	11.4
	ERBB2	3.4
	ERBB3	2.9
	Myc	2.1
**Downregulated (≥2-folds)**		
Apoptotic regulator	Bcl-2	−13.9

**Table 2:** Combination index (CI). Summary of fractional activity (Fa) at different points in the MCF7_DoxS cells and MCF7_DoxR cells. Cells were treated with different combinations of siRNA for 72 h and the combination index was calculated using Compusyn software.

Cell Type	siRNA Combination	CI/Fa = 0.5	CI/Fa = 0.75	CI/Fa = 0.9	CI/Fa = 0.95	CI/Fa = 0.97
MCF7_DoxS	STAT3 + β-catenin	1.19	1.20	1.21	1.21	1.22
	STAT3 + Notch-1	0.58	0.59	0.61	0.62	0.62
	β-Catenin + Notch-1	1.07	1.06	1.05	1.05	1.04
	STAT3 + β-catenin + Notch-1	0.69	0.68	0.67	0.67	0.66
MCF7_DoxR	STAT3 + β-catenin	1.11	1.08	1.06	1.06	1.06
	STAT3 + Notch-1	0.53	0.54	0.56	0.58	0.59
	β-Catenin + Notch-1	1.07	1.07	1.08	1.08	1.08
	STAT3 + β-catenin + Notch-1	0.50	0.52	0.53	0.54	0.55

## References

[1] McGuire S. (2016). World Cancer Report 2014. Geneva, Switzerland: World Health Organization, International Agency for Research on Cancer, WHO Press, 2015. Adv. Nutr..

[2] Ghoncheh M., Pournamdar Z., Salehiniya H. (2016). Incidence and Mortality and Epidemiology of Breast Cancer in the World. Asian Pac. J. Cancer Prev..

[3] Hanahan D., Weinberg R.A. (2011). Hallmarks of cancer: The next generation. Cell.

[4] Housman G., Byler S., Heerboth S., Lapinska K., Longacre M., Snyder N., Sarkar S. (2014). Drug resistance in cancer: An overview. Cancers.

[5] Vasir J.K., Labhasetwar V. (2005). Targeted drug delivery in cancer therapy. Technol. Cancer Res. Treat.

[6] Baudino T.A. (2015). Targeted Cancer Therapy: The Next Generation of Cancer Treatment. Curr. Drug Discov. Technol..

[7] Takebe N., Miele L., Harris P.J., Jeong W., Bando H., Kahn M., Yang S.X., Ivy S.P. (2015). Targeting Notch, Hedgehog, and Wnt pathways in cancer stem cells: Clinical update. Nat. Rev. Clin. Oncol..

[8] Li J., Wang Y., Zhu Y., Oupicky D. (2013). Recent advances in delivery of drug-nucleic acid combinations for cancer treatment. J. Control Release.

[9] Kc R.B., Thapa B., Valencia-Serna J., Aliabadi H.M., Uludag H. (2017). Nucleic acid combinations: A new frontier for cancer treatment. J. Control Release.

[10] Cardoso A., Trabulo S., Moreira J.N., Düzgüneş N., de Lima M.C.P. (2009). Targeted lipoplexes for siRNA delivery. Methods Enzymol..

[11] Tseng Y.-C., Mozumdar S., Huang L. (2009). Lipid-based systemic delivery of siRNA. Adv. Drug Deliv. Rev..

[12] Eroles P., Bosch A., Perez-Fidalgo J.A., Lluch A. (2012). Molecular biology in breast cancer: Intrinsic subtypes and signaling pathways. Cancer Treat. Rev..

[13] Cancer Genome Atlas N. (2012). Comprehensive molecular portraits of human breast tumours. Nature.

[14] Zang S., Chen F., Dai J., Guo D., Tse W., Qu X., Ma D., Ji C. (2010). RNAi-mediated knockdown of Notch-1 leads to cell growth inhibition and enhanced chemosensitivity in human breast cancer. Oncol. Rep..

[15] Yang Z., Cai J.H., Xie S.J., Li G.X., Song W.Q., Yan Q.H., Yan L., Zhang F. (2011). Therapeutic effects of signal transducer and activator of transcription 3 siRNA on human breast cancer in xenograft mice. Chin. Med. J..

[16] Zhang D., Fei F., Li S., Zhao Y., Yang Z., Qu J., Zhang X., Yin Y., Zhang S. (2017). The role of beta-catenin in the initiation and metastasis of TA2 mice spontaneous breast cancer. J. Cancer.

[17] Deng X., Apple S., Zhao H., Song J., Lee M., Luo W., Wu X., Chung D., Pietras R.J., Chang H.R. (2017). CD24 Expression and differential resistance to chemotherapy in triple-negative breast cancer. Oncotarget.

[18] Van Phuc P., Nhan P.L., Nhung T.H., Tam N.T., Hoang N.M., Tue V.G., Thuy D.T., Ngoc P.K. (2011). Downregulation of CD44 reduces doxorubicin resistance of CD44CD24 breast cancer cells. OncoTargetsTher..

[19] Minotti G., Menna P., Salvatorelli E., Cairo G., Gianni L. (2004). Anthracyclines: Molecular advances and pharmacologic developments in antitumor activity and cardiotoxicity. Pharmacol. Rev..

[20] Tassone P., Tagliaferri P., Perricelli A., Blotta S., Quaresima B., Martelli M., Goel A., Barbieri V., Costanzo F., Boland C. (2003). BRCA1 expression modulates chemosensitivity of BRCA1-defective HCC1937 human breast cancer cells. Br. J. Cancer.

[21] Taylor C.W., Dalton W.S., Parrish P.R., Gleason M.C., Bellamy W.T., Thompson F.H., Roe D.J., Trent J.M. (1991). Different mechanisms of decreased drug accumulation in doxorubicin and mitoxantrone resistant variants of the MCF7 human breast cancer cell line. Br. J. Cancer.

[22] AbuHammad S., Zihlif M. (2013). Gene expression alterations in doxorubicin resistant MCF7 breast cancer cell line. Genomics.

[23] Weihua Z., Lin Q., Ramoth A.J., Fan D., Fidler I.J. (2011). Formation of solid tumors by a single multinucleated cancer cell. Cancer.

[24] Sansone P., Bromberg J. (2012). Targeting the interleukin-6/Jak/stat pathway in human malignancies. J. Clin. Oncol..

[25] VanKlompenberg M.K., Bedalov C.O., Soto K.F., Prosperi J.R. (2015). APC selectively mediates response to chemotherapeutic agents in breast cancer. BMC Cancer.

[26] VanKlompenberg M.K., Leyden E., Arnason A.H., Zhang J.T., Stefanski C.D., Prosperi J.R. (2017). APC loss in breast cancer leads to doxorubicin resistance via STAT3 activation. Oncotarget.

[27] Kumawat K., Koopmans T., Gosens R. (2014). β-catenin as a regulator and therapeutic target for asthmatic airway remodeling. Expert Opin. Ther. Targets.

[28] Fan K., Li N., Qi J., Yin P., Zhao C., Wang L., Li Z., Zha X. (2014). Wnt/β-catenin signaling induces the transcription of cystathionine-γ-lyase, a stimulator of tumor in colon cancer. Cell. Signal..

[29] Teng Y., Wang X., Wang Y., Ma D. (2010). Wnt/β-catenin signaling regulates cancer stem cells in lung cancer A549 cells. Biochem. Biophys. Res. Commun..

[30] Deng Y., Pu X., Huang M., Xiao J., Zhou J., Lin T., Lin E.H. (2010). 5-Fluorouracil upregulates the activity of Wnt signaling pathway in CD133-positive colon cancer stem-like cells. Chin. J. Cancer.

[31] Bourguignon L.Y., Xia W., Wong G. (2009). Hyaluronan-mediated CD44 interaction with p300 and SIRT1 regulates beta-catenin signaling and NFkappaB-specific transcription activity leading to MDR1 and Bcl-xL gene expression and chemoresistance in breast tumor cells. J. Biol. Chem..

[32] Reedijk M., Odorcic S., Zhang H., Chetty R., Tennert C., Dickson B.C., Lockwood G., Gallinger S., Egan S.E. (2008). Activation of Notch signaling in human colon adenocarcinoma. Int. J. Oncol..

[33] Wang Z., Banerjee S., Li Y., Rahman K.W., Zhang Y., Sarkar F.H. (2006). Down-regulation of Notch-1 inhibits invasion by inactivation of nuclear factor-κB, vascular endothelial growth factor, and matrix metalloproteinase-9 in pancreatic cancer cells. Cancer Res..

[34] Wang Z., Li Y., Banerjee S., Kong D., Ahmad A., Nogueira V., Hay N., Sarkar F.H. (2010). Down-regulation of Notch-1 and Jagged-1 inhibits prostate cancer cell growth, migration and invasion, and induces apoptosis via inactivation of Akt, mTOR, and NF-κB signaling pathways. J. Cell. Biochem..

[35] Politi K., Feirt N., Kitajewski J. (2004). Notch in Mammary Gland Development and Breast Cancer. Seminars in Cancer Biology..

[36] Weijzen S., Rizzo P., Braid M., Vaishnav R., Jonkheer S.M., Zlobin A., Osborne B.A., Gottipati S., Aster J.C., Hahn W.C. (2002). Activation of Notch-1 signaling maintains the neoplastic phenotype in human Ras-transformed cells. Nat. Med..

[37] Diévart A., Beaulieu N., Jolicoeur P. (1999). Involvement of Notch1 in the development of mouse mammary tumors. Oncogene.

[38] Cho S., Lu M., He X., Ee P.L., Bhat U., Schneider E., Miele L., Beck W.T. (2011). Notch1 regulates the expression of the multidrug resistance gene ABCC1/MRP1 in cultured cancer cells. Proc. Natl. Acad. Sci. USA.

[39] Szklarczyk D., Morris J.H., Cook H., Kuhn M., Wyder S., Simonovic M., Santos A., Doncheva N.T., Roth A., Bork P. (2017). The STRING database in 2017: Quality-controlled protein-protein association networks, made broadly accessible. Nucleic Acids Res..

[40] Al-Hajj M., Wicha M.S., Benito-Hernandez A., Morrison S.J., Clarke M.F. (2003). Prospective identification of tumorigenic breast cancer cells. Proc. Natl. Acad. Sci. USA.

[41] Li W., Ma H., Zhang J., Zhu L., Wang C., Yang Y. (2017). Unraveling the roles of CD44/CD24 and ALDH1 as cancer stem cell markers in tumorigenesis and metastasis. Sci. Rep..

[42] Ricardo S., Vieira A.F., Gerhard R., Leitao D., Pinto R., Cameselle-Teijeiro J.F., Milanezi F., Schmitt F., Paredes J. (2011). Breast cancer stem cell markers CD44, CD24 and ALDH1: Expression distribution within intrinsic molecular subtype. J. Clin. Pathol..

[43] Mylona E., Giannopoulou I., Fasomytakis E., Nomikos A., Magkou C., Bakarakos P., Nakopoulou L. (2008). The clinicopathologic and prognostic significance of CD44+/CD24(-/low) and CD44-/CD24+ tumor cells in invasive breast carcinomas. Hum. Pathol..

[44] Ahmed M.A., Aleskandarany M.A., Rakha E.A., Moustafa R.Z., Benhasouna A., Nolan C., Green A.R., Ilyas M., Ellis I.O. (2012). A CD44(-)/CD24(+) phenotype is a poor prognostic marker in early invasive breast cancer. Breast Cancer Res. Treat..

[45] Marotta L.L., Almendro V., Marusyk A., Shipitsin M., Schemme J., Walker S.R., Bloushtain-Qimron N., Kim J.J., Choudhury S.A., Maruyama R. (2011). The JAK2/STAT3 signaling pathway is required for growth of CD44(+)CD24(-) stem cell-like breast cancer cells in human tumors. J. Clin. Investig..

[46] Tsou S.-H., Chen T.-M., Hsiao H.-T., Chen Y.-H. (2015). A critical dose of doxorubicin is required to alter the gene expression profiles in MCF-7 cells acquiring multidrug resistance. PLoS ONE.

[47] Wang X., Crowe P.J., Goldstein D., Yang J.L. (2012). STAT3 inhibition, a novel approach to enhancing targeted therapy in human cancers (review). Int. J. Oncol..

[48] Deng J., Miller S.A., Wang H.Y., Xia W., Wen Y., Zhou B.P., Li Y., Lin S.Y., Hung M.C. (2002). beta-catenin interacts with and inhibits NF-kappa B in human colon and breast cancer. Cancer Cell.

[49] Armanious H., Gelebart P., Mackey J., Ma Y., Lai R. (2010). STAT3 upregulates the protein expression and transcriptional activity of beta-catenin in breast cancer. Int. J. Clin. Exp. Pathol..

[50] Jin Y.H., Kim H., Ki H., Yang I., Yang N., Lee K.Y., Kim N., Park H.S., Kim K. (2009). Beta-catenin modulates the level and transcriptional activity of Notch1/NICD through its direct interaction. Biochim. Biophys. Acta.

[51] Hayward P., Brennan K., Sanders P., Balayo T., DasGupta R., Perrimon N., Martinez Arias A. (2005). Notch modulates Wntsignalling by associating with Armadillo/beta-catenin and regulating its transcriptional activity. Development.

[52] Jin S., Mutvei A.P., Chivukula I.V., Andersson E.R., Ramskold D., Sandberg R., Lee K.L., Kronqvist P., Mamaeva V., Ostling P. (2013). Non-canonical Notch signaling activates IL-6/JAK/STAT signaling in breast tumor cells and is controlled by p53 and IKKalpha/IKKbeta. Oncogene.

[53] Zhao C., Zhang M., Liu W., Wang C., Zhang Q., Li W. (2015). beta-Catenin knockdown inhibits pituitary adenoma cell proliferation and invasion via interfering with AKT and gelatinases expression. Int. J. Oncol..

[54] Ayyanan A., Civenni G., Ciarloni L., Morel C., Mueller N., Lefort K., Mandinova A., Raffoul W., Fiche M., Dotto G.P. (2006). Increased Wnt signaling triggers oncogenic conversion of human breast epithelial cells by a Notch-dependent mechanism. Proc. Natl. Acad. Sci. USA.

[55] Gariboldi M.B., Ravizza R., Molteni R., Osella D., Gabano E., Monti E. (2007). Inhibition of Stat3 increases doxorubicin sensitivity in a human metastatic breast cancer cell line. Cancer Lett..

[56] Xu J., Prosperi J.R., Choudhury N., Olopade O.I., Goss K.H. (2015). β-Catenin is required for the tumorigenic behavior of triple-negative breast cancer cells. PLoS ONE.

[57] Jaganathan H., Mitra S., Srinivasan S., Dave B., Godin B. (2014). Design and in vitro evaluation of layer by layer siRNAnanovectors targeting breast tumor initiating cells. PLoS ONE.

[58] Sakamoto K., Fujii T., Kawachi H., Miki Y., Omura K., Morita K., Kayamori K., Katsube K., Yamaguchi A. (2012). Reduction of NOTCH1 expression pertains to maturation abnormalities of keratinocytes in squamous neoplasms. Lab. Investig..

[59] Jiang G., Xiao X., Zeng Y., Nagabhushanam K., Majeed M., Xiao D. (2013). Targeting beta-catenin signaling to induce apoptosis in human breast cancer cells by z-guggulsterone and Gugulipid extract of Ayurvedic medicine plant Commiphoramukul. BMC Complement. Altern. Med..

[60] Qiagen. www.SABiosciences.com/pcrarraydataanalysis.php.

[61] Livak K.J., Schmittgen T.D. (2001). Analysis of relative gene expression data using real-time quantitative PCR and the 2(-Delta DeltaC(T)) Method. Methods.

[62] Rueden C.T., Schindelin J., Hiner M.C., DeZonia B.E., Walter A.E., Arena E.T., Eliceiri K.W. (2017). ImageJ2: ImageJ for the next generation of scientific image data. BMC Bioinform..

[63] Nishimura M., Jung E.-J., Shah M.Y., Lu C., Spizzo R., Shimizu M., Han H.D., Ivan C., Rossi S., Zhang X. (2013). Therapeutic synergy between microRNA and siRNA in ovarian cancer treatment. Cancer Discov..

